# Oral dextrose reduced procedural pain without altering cellular ATP metabolism in preterm neonates: a prospective randomized trial

**DOI:** 10.1038/s41372-020-0634-0

**Published:** 2020-02-26

**Authors:** Danilyn M. Angeles, Danilo S. Boskovic, John C. Tan, Wendy Shih, Erin Hoch, Dorothy Forde, Raylene M. Phillips, Andrew Hopper, Douglas D. Deming, Mitchell Goldstein, Giang Truong, Aprille Febre, Priscilla Pegis, Adrian Lavery, Munaf Kadri, Anamika Banerji, Iman Mousselli, Vora Farha, Elba Fayard

**Affiliations:** 1 Department of Basic Sciences, School of Medicine, Loma Linda University, Loma Linda, CA USA; 2 Department of Pediatrics, School of Medicine, Loma Linda University, Loma Linda, CA USA; 3 School of Public Health, Loma Linda Univeristy, Loma Linda, CA USA; 4 Loma Linda University Children’s Hospital, Loma Linda, CA USA; 5 School of Nursing, University of California, San Francisco, CA USA

**Keywords:** Translational research, Predictive markers

## Abstract

**Objective:**

To examine the effects of 30% oral dextrose on biochemical markers of pain, adenosine triphosphate (ATP) degradation, and oxidative stress in preterm neonates experiencing a clinically required heel lance.

**Study design:**

Utilizing a prospective study design, preterm neonates that met study criteria (*n* = 169) were randomized to receive either (1) 30% oral dextrose, (2) facilitated tucking, or (3) 30% oral dextrose and facilitated tucking 2 min before heel lance. Plasma markers of ATP degradation (hypoxanthine, uric acid) and oxidative stress (allantoin) were measured before and after the heel lance. Pain was measured using the premature infant pain profile-revised (PIPP-R).

**Results:**

Oral dextrose, administered alone or with facilitated tucking, did not alter plasma markers of ATP utilization and oxidative stress.

**Conclusion:**

A single dose of 30% oral dextrose, given before a clinically required heel lance, decreased signs of pain without increasing ATP utilization and oxidative stress in premature neonates.

## Introduction

Premature infants admitted to the neonatal intensive care unit (NICU) require up to several hundred procedures during their hospitalization [1]. Many of these are tissue-damaging procedures (TDPs) known to cause pain. It was previously shown that TDPs, such as tape removal, not only lead to signs of pain but also increase markers of adenosine triphosphate (ATP) degradation and oxidative stress [2]. Further investigations probed if interventions that appear to relieve pain also reduce biochemical markers of ATP degradation and oxidative stress. Oral sucrose is a commonly used analgesic that has been shown to significantly decrease pain scores when given prior to TDPs [3, 4], and was expected to also reduce markers of ATP degradation and oxidative stress. However, although 24% oral sucrose significantly reduced behavioral markers of pain, it significantly increased heart rate and biochemical markers of ATP degradation (hypoxanthine, uric acid) and oxidative stress (allantoin) over time [5].

Moreover, this effect was enhanced in neonates that were intubated or were receiving more than 30% FiO_2_ [6]. It, therefore, became necessary to look for an intervention, or a combination of interventions, which would decrease both behavioral and physiological markers of procedural pain, and not increase the biochemical markers of ATP utilization and oxidative stress.

## Method

The individual and additive effects of two commonly used interventions for procedural pain were tested. These interventions were (a) 30% oral dextrose (d-glucose), (b) facilitated tucking, and (c) a combination of 30% oral dextrose and facilitated tucking. Sweet solutions such as sucrose and dextrose are documented to reduce pain scores [4, 7, 8]. However, oral dextrose has the potential to decrease behavioral and physiological signs of pain while avoiding the metabolic costs of fructose, a key component of sucrose [9]. The adverse effects of oral sucrose include a reduction in ATP synthesis and phosphate depletion due to poorly regulated fructose metabolism [9, 10]. Facilitated tucking is the gentle positioning of preterm infants with arms and legs in a flexed, midline position close to the body, while either in a side-lying or prone position [11]. Facilitated tucking has been documented to reduce the behavioral and physiological signs of pain, as evidenced by a reduction in pain scores and heart rate [11, 12]. This study’s painful procedure is a clinically required heel lance, which refers to a puncture of the newborn’s heel for a blood glucose test using a specially designed lancet.

**Fig. 1:**
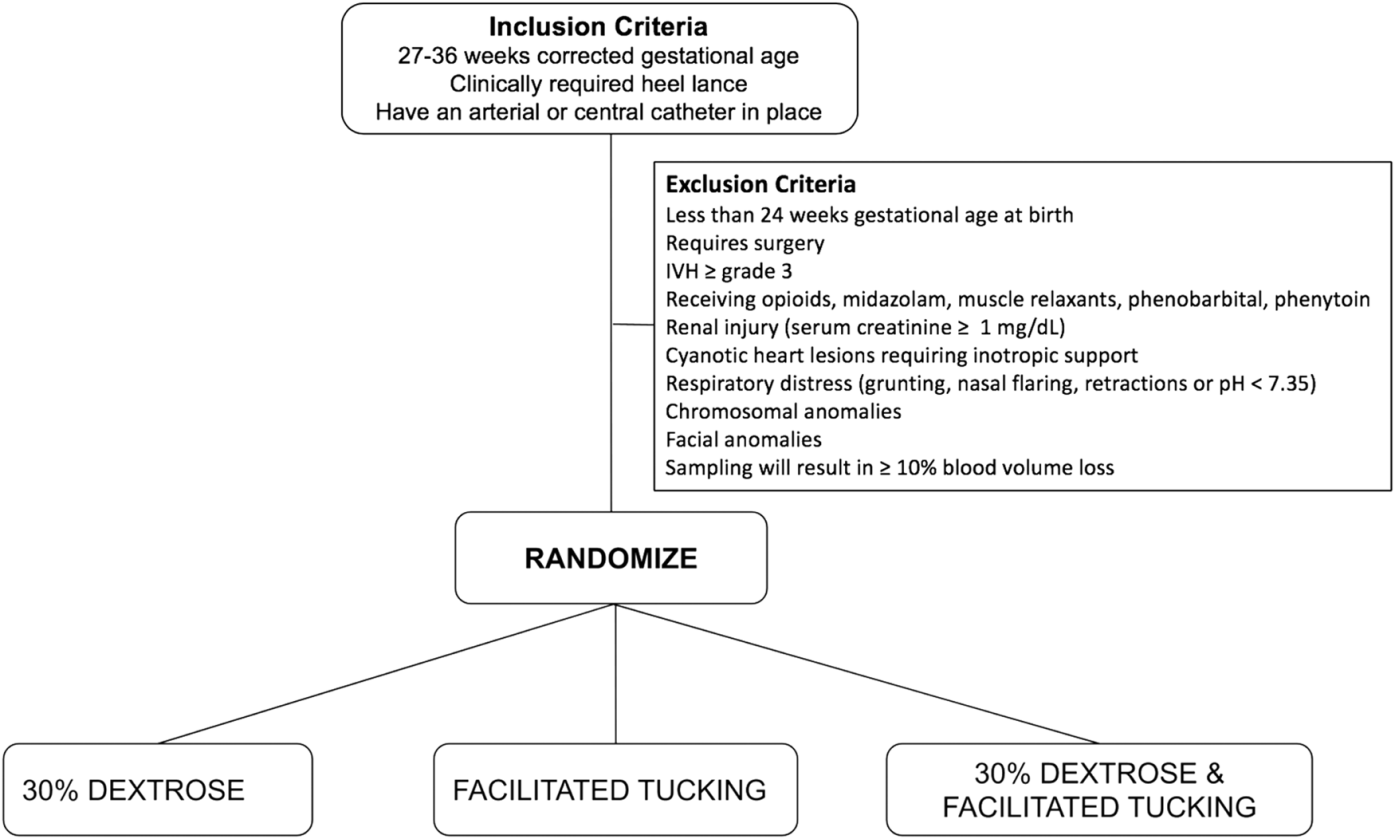
Study inclusion and exclusion criteria and experimental groups. Randomization procedure.

**Fig. 2:**
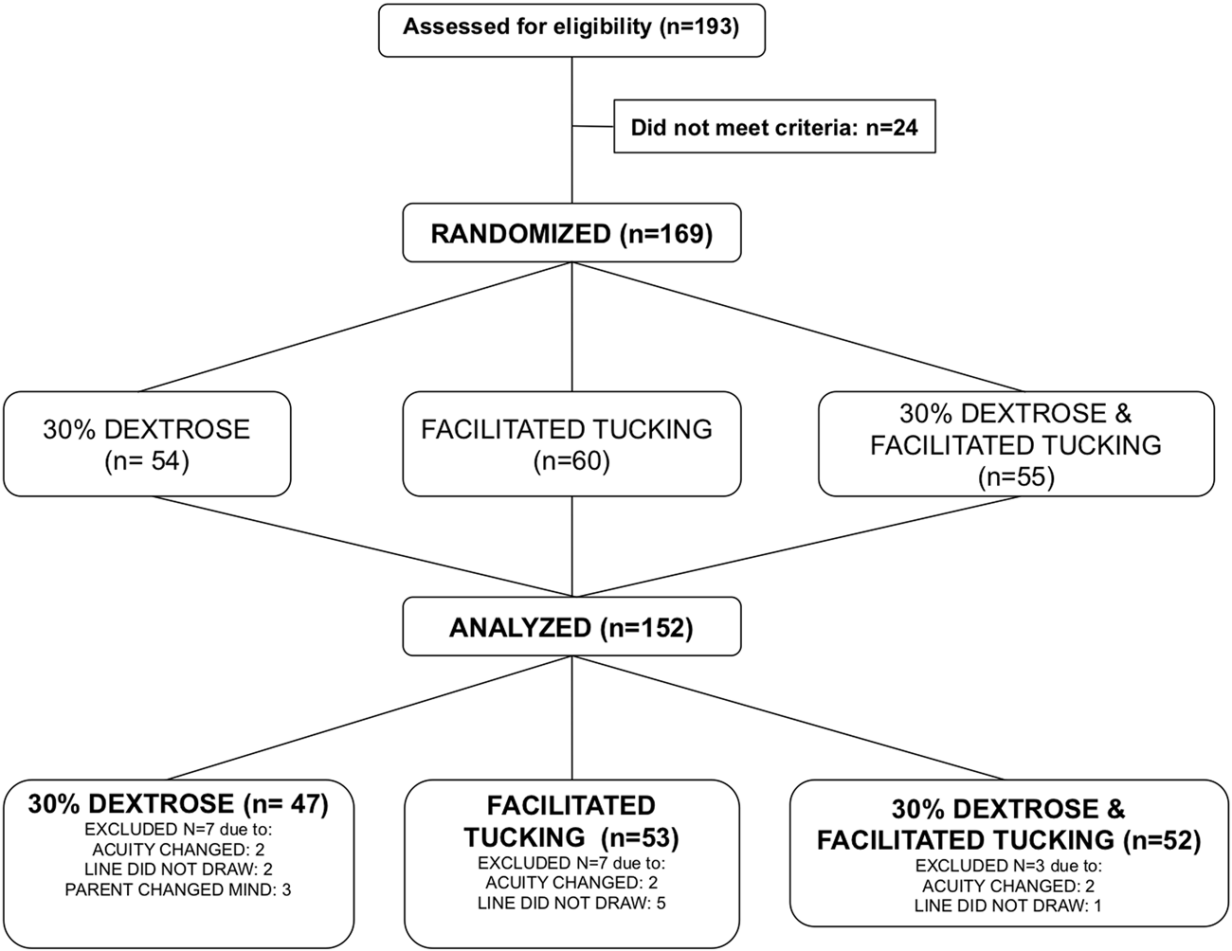
Flowchart of subject enrollment, randomization and analysis. Enrollment layout.

A prospective randomized study was conducted at Loma Linda University Children’s Hospital Neonatal Intensive Care Unit (LLUCH-NICU) from October 2014 to December 2018. The study protocol and informed consent documents were approved by the Loma Linda University Health Institutional Review Board. Families were approached for consent as soon as possible after birth. Figure 1 describes the study inclusion and exclusion criteria and the flowchart of subject enrollment. Subjects were premature infants born at >24 weeks gestational age (GA) who had an arterial or central catheter in place and clinically required heel lancing at 27–36 weeks corrected GA. Exclusion criteria included (1) requirement for surgery, (2) intraventricular hemorrhage (IVH) ≥ grade 3, (3) neonates on medications such as morphine, fentanyl, midazolam, muscle relaxants, phenobarbital, or phenytoin, (4) renal injury (plasma creatinine >1 mg/dl), (5) cyanotic heart lesions requiring inotropic support, (6) respiratory distress (increased work of breathing as evidenced by tachypnea, nasal flaring, chest retractions and grunting [13], (7) chromosomal anomalies, and (8) facial anomalies. To minimize the risk for hypovolemia, potential subjects were excluded if study sampling (along with clinically-related blood sampling) could potentially result in blood volume loss of more than 10%. Parents of premature infants who met the study criteria were approached for informed consent soon after birth. Once consent was obtained, subjects were randomized as described in Fig. 2. Randomization was performed by the research pharmacist using randomization tables generated by our statistician.

### Sample size determination

The sample size for the study was determined based on statistical power to detect changes in outcomes for the three intervention groups across time. Using a repeated-measures ANOVA (i.e., *F* test) based on a Type-I error rate of 5%, a total of 120 subjects (40 in each group) are needed to detect a small to moderate effect size of 0.25 (changes in pain scores and biochemical marker concentration) with 80% power. After accounting for an anticipated 10% attrition rate, a total of 132 subjects (44 in each group) would be needed.

**Fig. 3:**
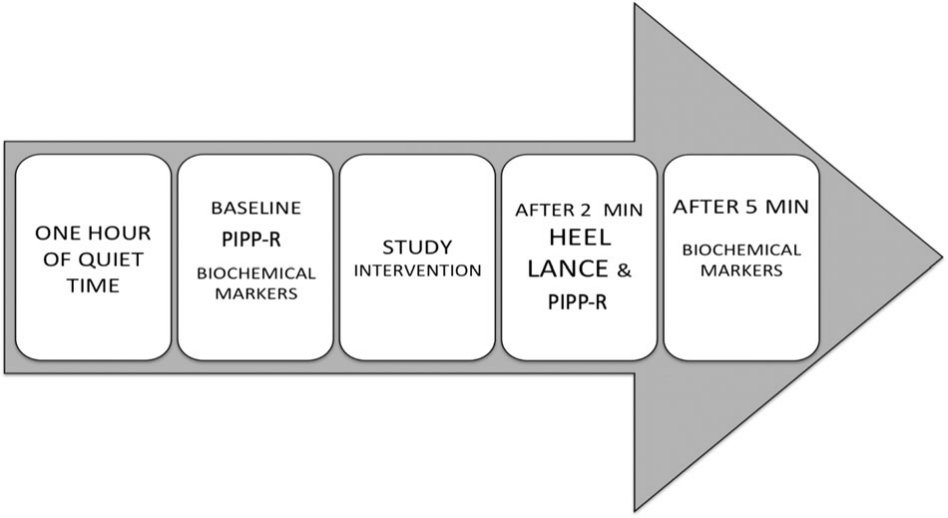
Data collection strategy and approach. Study procedure.

The flow of the study procedure is described in Fig. 3. Pain scores were measured at baseline (0 min) and at the time of the heel lance, and were determined on a scale of 0 through 21 using the premature infant pain profile-revised (PIPP-R) [14]. Biochemical plasma markers were measured at baseline (0 min) and 5 min after the heel lance. The “5 min” time period was chosen based on prior studies, demonstrating significant increases in plasma purines 5 min after heel lance, followed by purine levels below baseline 15–30 min after heel lance [5]. The dose of 30% dextrose, 0.5 mL/Kg was selected based on published studies on premature infants, showing this dose to be most effective in reducing signs of pain [8, 15–17]. Oral dextrose was prepared by our clinical pharmacist and was administered over 60 s via syringe to the anterior tongue 2 min prior to the heel lance followed by giving the subject a pacifier.

### Assessment of pain

We used the PIPP-R [14], an instrument that was designed to assess acute pain in preterm neonates. This tool has a physiological component that scores changes in oxygen saturation and heart rate as well as a behavioral component comprising facial expressions and behavioral state. Changes in facial expression include *brow lowering* or *brow bulge*, *eye squeeze*, and *nasolabial furrowing*. The facial expression score is based on the duration of observed expression (the longer the duration of brow bulge, the higher the score, up to a maximum score of 3 for each facial expression). A score of 6 or below indicates an absence of pain, while a score of 12 and above indicates moderate to severe pain [14].

### Measurement of biochemical markers of hypoxia, oxidative stress, and oxidative cell injury in plasma

Plasma markers were measured at baseline (“0” min) and 5 min after completion of heel lance. A total of 1.6 mL of blood (0.8 mL for each sampling period) was collected from an arterial or central catheter and placed in cooled plastic vials containing EDTA. Blood samples were centrifuged within 5 min to separate the plasma, which was stored at −80 °C. All samples were analyzed immediately or within 1 week of acquisition.

### Measurement of purines

Purine metabolites (hypoxanthine and uric acid) were measured as previously published by our laboratory [2, 5, 6]. Specifically, plasma was removed, transferred to separate Eppendorf tubes, and immediately centrifuged in Eppendorf 5702R (Pittsburgh, Pennsylvania) centrifuge, for 30 min at 18,000 × *g*. The supernatant was transferred to Microcon centrifugal filter devices (Millipore Corp, Bedford, Massachusetts), 200 μL per device, and spun for 90 min at 14,000 × *g*, 4 °C. The filtrate was removed, and 150 μL was transferred to an Eppendorf tube containing 1 × 10^−7^ mol of 2-aminopurine (internal standard). High-performance liquid chromatography (HPLC) (Waters 996 PDA, 715 Ultra Wisp Sample Processor; Millipore Corp) analysis was performed on the same day, or the tubes were frozen at −80 °C until analysis. Previous HPLC analysis of plasma demonstrated that purines remained stable with freezing [2, 5].

Three 45 μL injections were used for each sample onto a Supelcosil LC-18-S 15 cm × 4.6 mm, 5 μm column (SGE, Austin, Texas), with the following isocratic conditions: 50 mM ammonium formate buffer, pH 5.5, flow rate 1.0 mL/min. Hypoxanthine and uric acid were quantified by obtaining peak areas at appropriate retention times and wavelengths [5, 18]. Once the peak area of 2-aminopurine at ~10.8 min and 305 nm was determined, the area ratios of hypoxanthine and uric acid to 2-aminopurine were determined and converted to micromolar concentrations using standard curves. Samples were analyzed in triplicates, and values with coefficients of variation of <10% were included in the final analyses. The limits of detection for the purines are as follows: 1.58 μM hypoxanthine and 5.0 μM uric acid. The research technician that performed purine analysis was blinded to group assignment.

### Measurement of allantoin

Allantoin was measured in plasma as previously published by our laboratory [2, 5, 6] using an adaptation of the methods developed by Gruber et al. [19] and Pavitt et al. [20]. Plasma (50 μL) was transferred to an Eppendorf tube containing 5 × 10^−10^ mol internal standard (50 μL 10 μM [^15^N]-labeled allantoin). Spiked samples were simultaneously deproteinized and extracted by the addition of 100 μL of acetonitrile. These samples were then vortexed and centrifuged at 20,000 × *g*, 4 °C for 5 min, and the supernatant was dried under N_2_. After drying, 50 μL of MTBSTFA (i.e., N-methyl-N-tert-butyldimethylsilyltrifluoroacetamide) in pyridine (1:1 vol/vol) was added, and the derivatization reaction was facilitated by incubation at 50 °C for 2 h. The analysis was performed on Agilent 6890N Network GC System connected to an Agilent 5973 Inert Mass Selective Detector (both Agilent Technologies, Inc, Santa Clara, California). The separation was performed using an Agilent 122–5532G capillary column (25.7 m length, 0.25 mm internal diameter). Helium was used as the carrier gas at a flow rate of 1.5 mL/min. Derivatized product (1 μL) was injected in split mode (split 20:1, split flow 29.4 mL/min, total flow 33.8 mL/min). The initial column temperature was set at 100 °C and held at that temperature for 2 min; it was increased to 180 °C at a rate of 10 °C/min. The column was held at this temperature for 4 min and then increased to 260 °C at a rate of 20 °C/min. This temperature was maintained until the end of the run. Allantoin was quantified using selected ion monitoring mode with the 398.00 m/z ion monitored for allantoin and the 400.00 m/z for DL-allantoin-5-^13^C;1-^15^N. The ion abundance ratios (398.00/400.00) were converted to micromolar concentrations by the use of a standard curve. The research technician that performed allantoin analysis was blinded to group assignment.

### Statistics

Clinical and demographic characteristics were stratified by intervention group, summarized as count and percentages (%) for categorical characteristics, and mean and standard deviation (SD) for continuous characteristics. Categorical variables were compared with intervention groups using a *χ*^2^ test and continuous variables using a *t*-test.

Repeated-measures ANOVA was utilized to evaluate the effects of interventions on plasma purines and allantoin concentrations over time, with main effects of intervention group, time, and intervention group by time interactions. Separate models were fit for each outcome. The intervention effect was defined as a significant interaction effect between intervention group and time. Correlations between purines, allantoin, and biobehavioral markers (i.e., PIPP-R) were examined using Spearman’s rho. Statistical analyses for this study were generated using SAS software (version 9.4). Statistical significance was defined as *p* < 0.05.

## Results

### Subject demographics

As shown in Fig. 2, we obtained consent from 169 subjects who were randomized into three groups to receive (a) 30% oral dextrose alone: *n* = 54, (b) facilitated tucking alone: *n* = 60 or (c) 30% oral dextrose and facilitated tucking: *n* = 55, respectively, 2 min before a clinically required heel lance. Seventeen subjects were excluded after randomization due to reasons described in Fig. 2. As shown in Table 1, there were no significant differences in birth weight, GA, Apgar scores, and acuity scores (SNAPPE-II) between the three groups.

**Table 1:** Subject demographics and clinical characteristics.

Subject characteristics	30% dextrose *n* = 47	Facilitated tucking *n* = 53	30% dextrose and facilitated tucking *n* = 52	*P* value
EGA at birth (weeks)	28^4/7^ ± 3^2/7^	29^1/7^ ± 2^6/7^	29^2/7^ ± 2^4/7^	0.535
EGA at time of study procedure (weeks)	31^2/7^ ± 2^3/7^	31^0/7^ ± 4^3/7^	31^3/7^ ± 2^1/7^	0.746
Gender	Male: 30 (64%) Female: 17 (36%)	Male: 35 (66%) Female: 18 (34%)	Male: 28 (54%) Female: 24 (46%)	0.404
Apgar score, 0 min median (min, max)	5 (1, 9)	4 (0, 9)	5 (1, 9)	0.347
Apgar score, 5 min median (min, max)	8 (1, 9)	8 (1, 9)	8 (2, 10)	0.613
SNAPPE-II at time of study procedure	20.9 ± 16.2	21.2 ± 16.0	16.3 ± 14.0	0.256
Mode of oxygen delivery at time of study procedure	Spontaneous RA: 10 HFNC: 5 NCPAP: 23 NIPPV: 3 SIMV: 5 HFV: 1	Spontaneous RA: 9 HFNC: 12 NCPAP: 27 NIPPV: 3 SIMV: 0 HFV: 2	Spontaneous RA: 6 HFNC: 12 NCPAP: 25 NIPPV: 6 SIMV: 3 HFV: 0	0.204

### Effect of 30% dextrose with and without facilitated tucking on behavioral and physiological markers of pain

There were no significant differences in baseline pain scores between the three intervention groups (Table 2). There were also no significant differences in pain scores in response to heel lance between the three groups (Table 2). During the heel lance, the median PIPP-R score of subjects in the dextrose only group was 7 (min–max: 0, 15), median PIPP-R score of subjects in the facilitated tucking only group was 8 (min–max: 0, 17), and median PIPP-R score of subjects in the combination dextrose and facilitated tucking group was 8 (min–max: 0,16), *p* = 0.783) (Table 2). Heart rate and oxygen saturation during heel lance were not significantly different between the three groups from baseline to heel lance (*p* = 0.276 and *p* = 0.163, respectively) (Table 2).

**Table 2:** Behavioral, physiological, and biochemical signs of pain, ATP utilization, and oxidative stress.

Characteristics	30% dextrose *n* = 47	Facilitated tucking *n* = 53	30% dextrose and facilitated tucking *n* = 52	*P* value*
PIPP-R: median (min, max)				
Baseline	5 (0, 17)	5 (0, 14)	6 (0, 13)	0.124
Heel lance	7 (0, 15)	8 (0, 17)	8 (0, 16)	0.783
Heart rate, bpm				
Mean ± SD				
Baseline	162.78 ± 12.63	160.90 ± 19.10	167.02 ± 14.41	0.159
Heel lance	165.02 ± 13.73	165.08 ± 13.34	162.70 ± 29.06	0.965
Minimum O_2_ sat during heel lance (%), mean ± SD	94.44 ± 4.69	91.33 ± 7.28	92.10 ± 5.77	0.052
Uric acid				
Baseline	145.82 (70.96)	119.84 (51.14)	136.57 (64.94)	0.08
5 min	140.76 (73.11)	120.4 (49.48)	133.56 (65.72)	0.201
Hypoxanthine				
Baseline	1.35 (1.35)	1.35 (2.09)	1.37 (1.21)	0.739
5 min	1.33 (1.29)	1.44 (2.17)	1.28 (1.13)	0.927
Allantoin				
Baseline	29.24 (18.29)	31.95 (24.17)	26.31 (13.83)	0.642
5 min	27.83 (16.68)	30.72 (21.13)	26.78 (14.84)	0.75

*Bivariate comparisons.

### Effects of 30% dextrose with and without facilitated tucking on markers of ATP metabolism (purines) and oxidative stress (allantoin)

Oral dextrose with and without facilitated tucking did not increase plasma markers of ATP metabolism and oxidative stress (Table 2). Mean concentrations of hypoxanthine and uric acid decreased slightly over time or did not change significantly from baseline (Table 2, *p* = 0.98 and *p* = 0.07). We also found no significant increase in allantoin across the three intervention groups (*p* = 0.266), even among neonates with minimal pain response to heel lance.

## Discussion

We found that the use of 30% oral dextrose, with or without facilitated tucking, 2 min before a heel lance had no significant effect on plasma markers of ATP utilization and oxidative stress. This finding is in contrast to the effects of using 24% sucrose (SweetEase^TM^), which was shown to increase cellular energy utilization and oxidative stress [5, 6]. The difference may be due to the metabolism of the fructose moiety of sucrose. Although fructose and dextrose (d-glucose) have identical chemical formulas, their chemical structure is dissimilar, resulting in distinctions in absorption and metabolism. Unlike fructose, the metabolism of glucose is highly regulated and dependent on cellular energy demand. If the cellular need for energy is modest, glucose influx is lessened through a reduction in the density of glucose transporters (GLUT2) at the plasma membrane, and glucose is diverted away from glycolysis through allosteric inhibition of phosphofructokinase-1 [10].

In contrast, the cellular influx and metabolism of fructose occurs largely independently of cell’s energy needs, leading to substrate-dependent phosphate depletion, decreased ATP synthesis, and uric acid production [9, 21]. This biochemical reaction has been documented in the hepatocytes of children and adults [22]. It was also shown in premature neonates, where a single dose of oral sucrose given before a clinically required heel lance increased plasma markers of increased ATP degradation [5, 6]. These findings suggested that administration of oral sucrose may increase ATP utilization due to the biochemical cost of metabolizing fructose. Our current data suggest that the administration of oral dextrose does not have this effect on ATP utilization.

More recently, the effect of fructose on glucose metabolism was investigated in adipocytes from human Simpson-Golabi-Behmel syndrome [23]. In the presence of 5 mM glucose representing normal blood glucose concentration, fructose was shown to divert glucose metabolism away from glycolysis and oxidative phosphorylation, reducing ATP synthesis [23]. Glucose metabolism was also shown to be diverted away from glycogenesis, gluconeogenesis, ribose-phosphate synthesis, and nucleotide synthesis [23]. Instead, glucose was metabolized to lactate and diverted to serine oxidation glycine cleavage pathway (SOGC), a pathway utilized for lipogenesis and storage [23]. The diversion of glucose to the SOGC pathway resulted in lower ATP synthesis due to diminished mitochondrial energy metabolism [23]. It will be important to determine if this effect occurs in other cell types since fructose is also taken up and metabolized by skeletal muscle cells and renal cortical cells [24–26].

Presented results show that administration of 30% oral dextrose did not increase plasma allantoin levels, suggesting that oral dextrose has no significant effect on reactive oxygen species (ROS) formation. This finding is different from that previously observed using sucrose. A single dose of oral sucrose, given before a clinically required heel lance, significantly increased plasma allantoin concentration over time, in neonates whose pain increased minimally (<33% increase in PIPP scores) in response to the heel lance. Formation of ROS may be due to fructose-induced hyperuricemia. While uric acid is documented to be an antioxidant, it can also act as a potent prooxidant molecule [25]. However, ROS can also be formed when accessible carbonyls of aldehydes or ketones interact with basic amino groups of proteins, or with free hydroxyls found in lipids [27]. At normal pH and temperature, glucose molecules are found in the stable 6-member glucopyranose ring form, limiting aldehyde exposure, and reducing ROS generation [27]. However, fructose forms a 5-member fructofuranose ring with two axial hydroxymethyl groups, exposing reactive ketone moiety [27]. This fructose-generated ROS can lead to cellular damage, especially in the liver, as has been demonstrated in cultured hepatocytes [28] and in animal models [29].

Heel lance was documented to result in moderate pain, with pain scores ranging from 8.5 to 11.2 (PIPP) in neonates given sterile water and pacifier [4, 8, 30]. We showed that 30% oral dextrose with and without facilitated tucking can reduce the pain score by a minimum of 2–3 points (Table 2). However, the difference in pain scores between the three intervention groups was not statistically significant, suggesting that facilitated tucking with nonnutritive sucking may be as effective as 30% oral dextrose in decreasing signs of pain. This is especially important because the long-term effects of repeated administration of sweet solutions like sucrose and dextrose, specifically on organs such as the brain, liver, kidneys, and skeletal muscle, are still unclear. A study by Johnston et al. showed that repeated sucrose administration effectively decreased pain scores, but higher doses of sucrose (>10 doses per day) were associated with lower scores for motor development, vigor, alertness, and orientation at 36 and 40 weeks gestation [31]. It is not known whether repeated doses of 30% oral dextrose have similar effects. In addition, a recent study in neonatal mice showed that repeated exposure to sucrose led to significantly smaller white matter volumes (corpus callosum, stria terminalis, and fimbria) as well as a significantly smaller hippocampus and cerebellum [32]. Mice pups that received ten daily doses of sucrose, with nonpainful handling, had significantly poorer short-term memory in adulthood [33].

Moreover, the effect of sucrose or dextrose on the gastrointestinal system, starting with the mouth, has not been investigated. Does the administration of sweet solutions for pain alter the oral and intestinal microbiome of the premature infant? Because hospitalized neonates are exposed to 7–17 painful procedures per day [1], leading to potential administration of repeated doses of oral sucrose or dextrose per day, it is important to examine the effect of oral sucrose or oral dextrose on the development of the gastrointestinal microbiome.

This study’s limitations are largely due to subjects’ GAs, illness severity, and treatment dosage. (1) The mean corrected GA of subjects at the time of study was 31 ± 2 weeks, making the findings not generalizable to neonates that are <29 weeks gestation. (2) Enrolled subjects were clinically stable, as demonstrated by the mean SNAPPE-II score of 17–21, to reduce the effects of illness or tissue hypoxia on purine and allantoin levels. This reduces the applicability of findings to critically ill preterm neonates, considered to have SNAPPE-II scores of 37 and above [34]. An appropriately effective analgesic for critically ill preterm neonates is still not known. (3) This study examined the effects of a *single dose* of 30% oral dextrose with and without facilitated tucking. Effects of prolonged repeated doses of dextrose on ATP metabolism, oxidative stress, and pain relief are unknown. In addition, effects of sweet solutions on other biochemical markers, such as markers of inflammation, are not known. Further prospective, randomized controlled trials that examine short and long-term effects of 30% oral dextrose are needed.

## Conclusions

Our findings show that 30% dextrose is an effective analgesic for preterm neonates experiencing heel lance. Because 30% dextrose, with or without facilitated tucking, decreased signs of pain without altering cellular energy metabolism, it may be a better nonpharmacologic intervention for procedural pain in premature neonates than interventions containing fructose (such as sucrose). Long-term cellular and organ effects of sweet solutions given during the neonatal period are not yet well understood in humans. Further studies are required to investigate these ubiquitously administered analgesics.
